# A Web-Based Intervention Based on Acceptance and Commitment Therapy for Family Caregivers of People With Dementia: Mixed Methods Feasibility Study

**DOI:** 10.2196/53489

**Published:** 2024-04-04

**Authors:** Golnaz. L Atefi, Rosalia J M van Knippenberg, Sara Laureen Bartels, Andrés Losada-Baltar, María Márquez-González, Frans R J Verhey, Marjolein E de Vugt

**Affiliations:** 1 Department of Psychiatry and Neuropsychology Alzheimer Centrum Limburg Maastricht University Maastricht Netherlands; 2 Departamento de Psicología Facultad de Ciencias de la Salud Universidad Rey Juan Carlos Madrid Spain; 3 Biological and Health Psychology Department School of Psychology Universidad Autónoma de Madrid Madrid Spain

**Keywords:** acceptance and commitment therapy, ACT, psychological flexibility, behavior change, theory-guided eHealth, web-based intervention, supported self-help, family caregivers, dementia

## Abstract

**Background:**

Acceptance and commitment therapy (ACT), as an empirically based third-wave cognitive behavioral therapy, has shown promise in enhancing well-being and functioning across diverse populations. However, in the context of caregiving, the effect size of available ACT interventions remains at best moderate, sometimes accompanied by high dropout rates, highlighting the need for more effective and feasible intervention designs.

**Objective:**

The objective of our study was to evaluate the feasibility and acceptability of a fully online ACT program designed for family caregivers of people with dementia. This study aimed to boost psychological flexibility and support caregivers, enabling them to realize and prioritize their own life values alongside their caregiving responsibilities.

**Methods:**

A mixed methods feasibility study using an uncontrolled pretest-posttest design was conducted. This intervention included a 9-week web-based self-help program based on ACT incorporating collaborative goal setting and weekly web-based motivational coaching for family caregivers of people with dementia. This study involved 30 informal caregivers recruited through memory clinics and social media platforms in the Netherlands and received approval from the Medical Ethics Committee of the Maastricht University Medical Center+ (NL77389.068.21/metc21-029).

**Results:**

A total of 24 caregivers completed the postintervention assessment, indicating a high adherence rate (24/29, 83%). Caregivers reported positive feedback regarding collaborative goal setting, but some found challenges in implementing new skills due to their own habitual responses or the unpredictable context of dementia caregiving. Personalizing the intervention based on individual value preferences was highlighted as beneficial.

**Conclusions:**

Compared to other web-based self-help ACT interventions for family caregivers, this intervention showed a high adherence and sufficient level of feasibility, which underscores the use of personalization in delivering web-based interventions. Moreover, the potential of this ACT-based intervention for family caregivers of people with dementia was demonstrated, suggesting that further research and a larger-scale controlled trial are warranted to validate its effectiveness.

**International Registered Report Identifier (IRRID):**

RR2-10.1136/bmjopen-2022-070499

## Introduction

### Background

The number of people with dementia is predicted to double every 20 years, which will lead to a corresponding rapid increase in the number of family caregivers [1]. Family care increases the quality of life of people with dementia and reduces formal care costs, thus making a substantial contribution to dementia care management. This is noteworthy as most people with dementia rely on a range of crucial and unpaid support from family caregivers [2]. Although the experience of caregiving might be fulfilling and positive, research also shows that, with advancing dementia, the need for care can become increasingly time-intensive, stressful, and more complex, posing a risk to the overall well-being of caregivers. In the context of dementia, family caregivers may experience inevitable and long-lasting changes due to the progressive nature of the condition and the increasing dependence of the person with dementia on their caregivers.

Importantly, the adverse effects of caregiving can be addressed and improved through a wide range of psychological interventions [3-6]. However, the long-term care situation and associated (inevitable) changes in caregivers’ lives underscore the importance of acceptance-based interventions that focus on developing skills to effectively address the management of maladaptive thoughts and emotions and acceptance of ongoing changes [7]. Specifically, the ways through which caregivers perceive and respond to internal (ie, thoughts and feelings) and external (ie, environmental) stressors are significant predictors of negative outcomes in this population [8,9]. Subsequently, acceptance and commitment therapy (ACT), as an empirically based third-wave cognitive behavioral therapy, might be particularly noteworthy due to its scalable focus on promoting psychological flexibility [10]. From the ACT perspective, psychological flexibility refers to efficient functioning in the presence of difficult experiences and is achieved via 6 interrelated core processes. These processes include openness to internal experiences (ie, acceptance), defusing from thoughts (ie, cognitive defusion), being in the present moment and aware of oneself and others (ie, mindfulness), having a distinct perspective on internal experiences (ie, self as context), identifying meaningful action qualities that can be connected with bringing purpose and motivation (ie, values), and active engagement and behavioral action aligned with values (ie, committed action) [11]. Hundreds of randomized controlled trials (RCTs) have demonstrated the effectiveness of ACT in improving overall well-being in the general population and in people with mental or somatic health problems [6,12].

### ACT for Family Caregivers of People With Dementia

In the context of dementia caregiving, ACT shows promise in promoting acceptance of change and increasing willingness to take meaningful actions, leading to improved psychological flexibility and better overall functioning in caregivers [13-15]. Several RCTs have demonstrated that face-to-face individual ACT interventions provided by trained therapists result in a significant reduction in depression and anxiety in family caregivers of people with dementia compared to control groups [13,15,16]. Furthermore, non-RCT ACT studies have also shown promise in supporting family caregivers of people with dementia through modalities such as telephones [17], videoconferencing [18], group settings [19], or web-based self-help modules [20,21].

### eHealth Adaptations for Family Caregivers

In recent years, technological advancements have facilitated the development and adaptation of a wide range of acceptable and promising psychological programs, from face-to-face to eHealth (ie, the use of internet to promote well-being) [4,22,23]. In particular, web-based self-help interventions are cost-effective and accessible approaches that reduce the significant involvement of health care professionals, allowing caregivers to complete the intervention on their own time using computers, tablets, or mobile devices. Web-based self-help interventions provide materials such as modules, text, and videos, enabling users to navigate the program at their own pace [24].

However, the effect sizes of the available interventions are still at best moderate [3] with a high dropout rate [20], indicating the need for more effective intervention designs [15]. Furthermore, in the context of caregiving, family caregivers of people with dementia are often older adults. The potential lower digital literacy and preference for traditional face-to-face psychological support, coupled with implementation limitations, make them the subgroup of caregivers who most frequently report challenges related to accessing and using eHealth interventions [4]. Thus, in the context of eHealth, guided self-help interventions including “minimal contact” might be a promising approach for this population. In a “minimal contact” approach, health care professionals (eg, coaches) are involved for nontherapeutic purposes and mainly for periodic check-ins, teaching participants how to use the digital tools, and provision of initial rationales [23,25].

### Personalizing Self-Help Interventions From the ACT Perspective

Personalizing interventions is an important and effective strategy to increase adherence and prevent dropout rates, making trials, including RCTs, more feasible, acceptable, and effective [26]. One effective and efficient approach to personalization is collaborative goal setting, in which individuals, together with health care professionals, set specific goals based on their own needs and resources before the intervention, leading to increased motivation and, ultimately, behavior change [27]. From the ACT perspective, there is a distinction between goals and value-based choices and their impact on (long-term) behavior change. Goals are typically external and have a clear end point that can be achieved or completed. In contrast, values are meaningful qualities that cannot be obtained or finished but rather help set meaningful goals and guide long-term patterns of behavior. Thus, acknowledging personal values and nesting specific goals underneath them is more likely to drive effective behavior change in the long term [11]. Engaging in value-based activities in the context of caregiving has a positive association with emotional well-being [28] and a negative association with distress [16].

To our knowledge, collaborative goal setting based on caregivers’ values has not yet been explored within the context of web-based ACT self-help interventions. Gaining further insights into the feasibility of web-based ACT interventions for family caregivers of people with dementia might contribute to decisions related to the implementation of ACT interventions in clinical practice, facilitating intervention refinements and, ultimately, leading to the design and development of more acceptable, effective, and sustainable interventions for future large-scale controlled trials.

### This Study

Despite the growing literature on ACT and the efficacy of eHealth interventions in family caregivers, there is a limited focus on understanding the specific challenges and opportunities of web-based self-help interventions for this population [29]. To date, few studies have used supported or personalized self-help ACT to address the specific needs of family caregivers. The aforementioned studies, although promising [21], showed a high dropout rate [20], or the intervention did not prove highly effective [30]. Therefore, in response to the need for a more efficient intervention design for family caregivers of people with dementia, this pilot trial aimed to use a mixed methods assessment approach (qualitative and quantitative data) to examine the feasibility and acceptability of the ACT for informal caregivers of people with dementia intervention. This web-based guided intervention is designed for community-based family caregivers of people with dementia through web-based ACT modules, collaborative goal setting based on individuals’ personal values before the intervention, and minimal-contact motivational coaching during the intervention. Subsequently, this study aimed to address the following research question: what are the practical and conceptual barriers and facilitators influencing the feasibility and acceptability of this guided web-based intervention?

## Methods

### Overview

This study was a pilot trial with a mixed methods approach, a baseline assessment, a 9-week web-based intervention embedded with web-based weekly motivational coaching, and a postintervention assessment. This study was reported according to the guidelines presented in the CONSORT (Consolidated Standards of Reporting Trials) checklist [31]. The CONSORT checklist is presented in Multimedia Appendix 1 [32]. The complete design is described in the protocol [33], and the methodological details relevant to this feasibility and acceptability study are presented in the following sections.

### Participants

A sample size of 30 participants is considered to be sufficient to enable a reasonable calculation of the key factors relevant to feasibility (eg, attrition rates) and provide useful information required for recommending a larger controlled trial [34,35]. Therefore, 30 family caregivers of people with dementia were recruited for this study.

Eligible family caregivers were recruited sequentially from May 2022 to June 2023. The following inclusion criteria were applied: (1) adult caregivers (aged ≥18 y), (2) self-identified primary family caregivers of a person with a diagnosis of dementia, (3) caring for the care recipient for at least 3 hours per week for at least 3 months, (4) internet and tablet or computer accessibility in the household, and (5) consent to participate. Family caregivers were excluded if (1) they indicated cognitive difficulties or disorders in their medical record (based on self-report) or (2) they had undergone psychotherapy or psychopharmacological treatment during the previous 3 months.

### Recruitment Procedure and Screening

**Figure 1:**
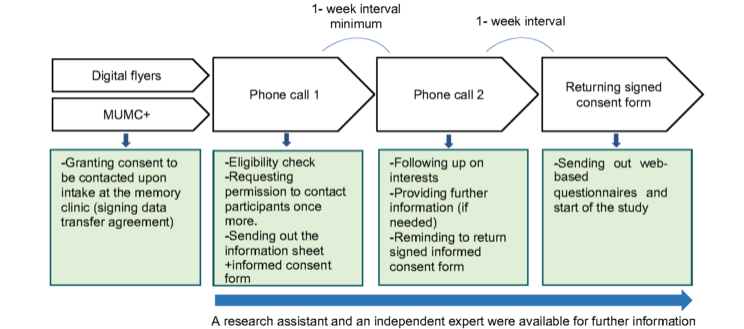
Recruitment procedure. MUMC+: Maastricht University Medical Center+.

Family caregivers were recruited from referrals by clinicians (eg, psychiatrists or psychologists) at the memory clinic of the Maastricht University Medical Center+ in the Netherlands. Furthermore, recruitment took place using printed or web-based flyers and website posts by patient and caregiver support organizations in the Netherlands, the Dutch Alzheimer Association, and local mental health institutions. Information about the study as well as a self-addressed stamped envelope for returning the informed consent form were provided to all the participants via post. When the research team received the signed informed consent form (in paper format), the study officially started. The process of screening and recruitment is shown in Figure 1, and further details on the recruitment procedure can be found in the study protocol [33].

### Intervention

#### Overview

The guided web-based intervention followed a written protocol [33]. As a general structure, each week, participants first completed a web-based module and then received a telephone or video call from a motivational coach. Participants were instructed to complete consecutive steps, including (1) a 50-minute web-based video call at baseline with a motivational coach for value-based goal setting, (2) 9 self-help web-based ACT modules (20-30 min each), and (3) an approximately 20-minute weekly web-based video call with a motivational coach for 9 weeks. In addition, participants had the flexibility to extend the duration of the intervention and complete the 9 modules and coaching sessions in 12 weeks. Contact with the coach took place in individual sessions.

#### Collaborative Goal Setting

Collaborative goal setting [36] was chosen to personalize the guided web-based intervention. During the individual value-based goal-setting session with the motivational coach, each participant set one value-based personalized goal that was specific, measurable, attainable, realistic, and time-orientated (SMART) [37]. Individuals could select from a predefined list of value-based actions or write goals in their own words (Multimedia Appendix 2). The list was prepared based on the ACT model and an adapted version of the valued living questionnaire for dementia caregiving [28,29,38]. Specific value-based actions with clear steps were defined during the collaborative goal-setting session and were embedded into future activities within the intervention. The steps of collaborative goal setting are shown in Multimedia Appendix 3.

#### Web-Based Self-Help Modules

Details about the intervention can be found elsewhere [33]; in brief, caregivers focused on 1 of the 6 ACT components (ie, acceptance, cognitive defusion, mindful attention, self as context, value, or committed action) in each module, gradually covering all the core principles of psychological flexibility. The 9 self-help web-based modules were released weekly consisting of a standard structure including a brief introductory text, a short video, an ACT-related metaphor, and content-oriented assignments. Module 1 provided a short introduction to ACT and a program overview. In module 2, “Creative Hopelessness” was introduced to reflect on the dysfunctionality of avoidance strategies for difficult thoughts and feelings in life. Modules 3 to 8 reviewed each of the 6 ACT components in detail. Finally, session 9 was a recap of all ACT components incorporating strategies for relapse prevention in everyday life (Multimedia Appendix 4). In addition, ACT metaphors were embedded in the content of the modules. For example, the boomerang metaphor was used for avoiding unpleasant feelings. The concept is that the more one attempts to throw the metaphorical boomerang away, the more forcefully it will eventually return [39].

After completing a module, caregivers received an automated email notification from the website reminding them of the availability of the next module. Access to the modules was sequential, requiring completion in a specific order rather than allowing access to all modules at once. Further details on the content of the website are presented in Table 1.

**Table 1:** Overview of the 9 modules included in the intervention.

Module	Title	ACT^a^ strategy	Example of the exercise
1	Introduction	The introduction of ACT and the content of the program consists of modules with videos explaining ACT components accompanied by metaphorical images and exercises to enhance personal resilience.	“You are now invited to answer these two questions for yourself: Where do you want to go with your life (as a person, privately and in your work)? What stands in your way?”
2	Creative hopelessness	Exploring creative hopelessness strategies among informal caregivers for navigating unpleasant thoughts and feelings.	“Share three personal examples of how you prevent unpleasant feelings, employing strategies like prevention, avoidance, and reduction. This exercise fosters self-awareness about your functioning in challenging situations.”
3	Acceptance	Recognizing caregivers’ potential struggles with negative emotions, this module involves fostering acceptance by making room for unpleasant feelings.	“This exercise encourages reflection on personal pain, strategies for coping, and resulting suffering. Identify specific instances of pain, whether thoughts, feelings, physical sensations, or situations.”
4	Defusion	Addressing the tendency of individuals to take their thoughts seriously, the focus is on defusion—encouraging individuals to distance themselves from difficult thoughts.	“Write a negative self-view, e.g., ‘I’m not good enough,’ then rephrase it as ‘I have the thought that I am not good enough.’ Notice the difference between directly identifying with the thought and acknowledging it as a passing idea.”
5	Self as context	Acknowledging the tendency of individuals to define their self-image based on perceived expectations, the emphasis is on the self as context—creating room for individuals to be themselves and fostering flexibility in their self-image to alleviate stress.	“In ACT, your self-image is like wearing a tailor-made suit that may not fit your current feelings. Reflect on times this felt restrictive and consider less uncomfortable suits you’ve worn. Explore a more flexible approach and think of the benefits of taking them off for a moment.”
6	Mindfulness	Recognizing that excessive focus on the past or future may not always be helpful, the emphasis is on the here and now—encouraging individuals to pay sufficient attention to the present moment, the only time when we can truly live, act, and experience.	“Reflecting on past and future often overshadows the present; embracing the moment enhances life satisfaction. Losing the present in thoughts might result in missing the richness of the present experience. Try to recognize an example of this in your own life.”
7	Value	Recognizing the significance of acknowledging what truly matters in one’s life, the focus is on values—actively encouraging individuals to ask whether their values are sufficiently present in their lives and put them into practice.	Value-sorting task: “Identify core life values using a set of cards, categorizing them as ‘Very Important,’ ‘Important,’ or ‘Not Important,’ and then prioritizing the top 10 values to actively invest in.”
8	Committed action	Emphasizing the facilitation of a more meaningful life, the focus is on committed action—encouraging individuals to define concrete and feasible actions toward their values, actively invest in them, and translate them into value-based actions.	“Take practical steps and reflect on each significant value and ask, ‘What is the smallest, easiest step I can take in the next 24 hours to align my life with that value?’ Ensure specificity and feasibility in these actions for practical implementation.”
9	Psychological flexibility	The concluding module highlights that cultivating psychological flexibility through the practice of the 6 core skills enables individuals to address problems differently and with greater flexibility, enriching their lives in ways that hold personal value.	“You were introduced to all the different ACT components. We invite you to answer the initial questions from Module One again: Where do you want to go with your life (as a person, privately and in your work)? What stands in your way?”

^a^ACT: acceptance and commitment therapy.

#### Weekly Coaching

A trained research assistant with experience in intervention coaching was appointed from Maastricht University to fulfill the role of the motivational coach. As part of the weekly coaching process, participants were encouraged to complete the weekly module before scheduling a video call. During the weekly coaching, the coach inquired about the participants’ overall experience with the web interface (ie, whether any technical or other issues occurred), how goal attainment was progressing (ie, value-based actions defined during the collaborative goal setting), and whether they experienced a (positive or negative) change in their motivation (Multimedia Appendix 5). Weekly coaching was not intended as a therapeutic function but rather to provide technical support and motivate participants to follow the web-based self-help modules (ie, adherence) and stay engaged with their value-based goals. Thus, goal attainment and module completion were not mandatory before coaching took place.

### Sampling and Intervention Quality

Data were collected using the Castor web-based platform provided by Maastricht University. Intervention integrity was monitored independently by experts. Several types of monitoring visits were conducted by the Clinical Trial Center Maastricht for the purpose of quality and control at the beginning of the study (ie, site initiation visit); during the course of the study (ie, interim monitoring visits); and at the end of the study, when participants had completed the study and all data had been collected (close-out visit) [33].

Quantitative and qualitative data were used to inform the feasibility and acceptability of the intervention for the participants and coach. The number of referrals, number of eligible participants willing and not willing to take part, reasons for declined participation or dropout after signing the informed consent form and before starting the intervention (if provided), and amount of time spent on recruiting 30 participants were monitored during recruitment. Quantitative and qualitative data on the intervention procedure were collected to inform use logs and interaction with the web-based modules. Barriers (eg, technical problems or difficulties) to user engagement were qualitatively collected during weekly coaching. To distinguish between intervention rejections and dropouts, we defined dropout as attrition following the start of the intervention (ie, those participants who attended at least one session of the intervention but discontinued it) [26]. Reasons for dropout after starting the intervention (if provided), attrition rate of weekly coaching sessions, and ACT module completion were also recorded.

### Assessment

#### Overview

This study specifically focused on feasibility and acceptability aspects. The preliminary efficacy and changes in psychological outcomes will be reported elsewhere. Further details on the measures of preliminary efficacy are described elsewhere [33].

#### End-of-Module Questionnaire

Following the previous study, the research team developed the end-of-module questionnaires [40] with the goal of gaining additional insights into the feasibility and acceptability of each module. Subsequently, content comprehension was assessed directly after completion of each module via a feedback questionnaire. This questionnaire included 3 items (ie, “I found today’s module useful,” “I have experienced the content of the modules as stressful,” and “I can apply the content of today’s modules in my daily life”) rated on a Likert scale ranging from 1=*strongly disagree* to 7=*strongly agree* on perceived usefulness, stressfulness, and applicability of each module to one’s daily life.

#### Goal Attainment

The level of goal achievement, as well as qualitative feedback on the feasibility and acceptability of goal achievement, was collected on a weekly basis during coaching. Goal attainment scaling was mapped on a prespecified ordinal scale, and the number of attainment levels ranged from −3 to +2. Each SMART goal was set at different layers as an “action list” including different levels of goal attainment. Level “0” was set as the “expected” level, and the rest of the levels were defined by a possible change in goal attainment. Any progress from the “expected level” was scored with “+1” as the “better than expected” level or “+2” as the “much better than expected” level. Deterioration in goal attainment was scored with “−1” as the “improvement but less than expected” level. The “−2” score was assigned to the “current” level and addressed “no change” from the goal-setting day, and “−3” referred to the “much less than expected” level in goal attainment. Setting an in-between “−2” score as the “current” level was considered to prevent floor effect and capture deterioration from the “current” individuals’ state [37]. During collaborative goal setting at baseline, each caregiver defined and clarified 5 layers of their SMART goals (from −3 to +2).

#### The Program Participation Questionnaire

Further insights into the usability, clarity, and acceptability of the intervention for family caregivers was obtained during a postintervention semistructured interview using the Program Participation Questionnaire (PPQ) [40]. The PPQ consisted of 26 items scored on a Likert scale ranging from 1=*strongly disagree* to 7=*strongly agree* focusing on 3 main areas, including the applicability of the intervention in everyday life, feasibility, usability, acceptability, and content quality and quantity (Multimedia Appendix 6). Furthermore, the number of log-ins to the modules and feature use were collected and compared with self-reported data. The feasibility and perceived experience of the coach was evaluated using a brief 6-item coach questionnaire focusing on the intervention’s usability and relevance for the coach, general perceived experience, the program’s positive and negative aspects, and suggestions for improvements (Multimedia Appendix 7).

#### Demographic Information

During the baseline assessment, family caregivers completed a demographics questionnaire providing information about their age, sex, level of education, living situation (ie, whether the caregiver and the person with dementia lived together or independently), hours of caregiving per week, type of kin relationship with the person with dementia, years since diagnosis, and dementia type.

### Data Analysis

The PPQ was analyzed quantitatively and qualitatively. Due to the lack of external criteria to properly define feasibility [41], median scores as a conventional strategy were defined as determinants of the overall feasibility, usability, and acceptability [40,42]. This approach to evaluating feasibility was also previously used in a Delphi study [43]. The overall PPQ scores ranged from 26 to 182. The median score of 130 was deemed as the cutoff and, thus, “acceptable feasibility” [40]. Mean item scores (range 1-7) of <5 (“slightly agree”) were considered as having potential for improvement. Participants further reflected qualitatively on their scores, and their reflections were audio recorded and transcribed verbatim. The deductive content analysis was conducted by authors GLA and RVK using field notes to interpret the quantitative scores on the PPQ [42,44]. In addition, to evaluate whether the intervention components aligned with the specific needs of the target group [45], field notes were classified and deductively coded into four main categories to address the intervention components: (1) overall experience and suggestions, (2) goal setting and value identification, (3) web-based self-help ACT modules, and (4) coaching. Furthermore, the self-report acceptability questionnaires at the end of each module were summarized in SPSS (IBM Corp) using descriptive statistics. Finally, web feature use and the number of log-ins were collected and subsequently compared with self-reported data. Data from the weekly coaching sessions were incorporated to complement the log data and the postintervention semistructured interviews.

### Ethical Considerations

The study was approved by the Medical Ethics Committee of the Maastricht University Medical Center+ (NL77389.068.21/metc21-029). All participants provided informed consent, and the rigorous protection of privacy and confidentiality of participants was safeguarded throughout the study. Data were anonymized when applicable to safeguard participant identities. As a token of appreciation, participants received a €25 (US $27.13) gift card for their involvement.

## Results

### Overview

**Figure 2:**
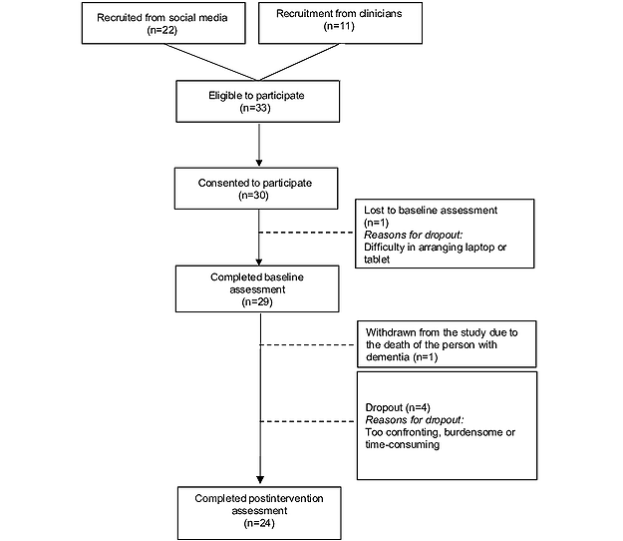
Participant flow.

A total of 33 family caregivers were deemed eligible to participate in the study, of whom 30 (91%) provided informed consent and 24 (73%) completed the postintervention assessment (Figure 2).

In total, 3 eligible caregivers declined to sign the informed consent form citing the extra burden on their caregiving responsibilities (n=2, 67%) and the admission of the person with dementia to a nursing home (n=1, 33%) as reasons for their decision. Of the 29 participants who initiated the intervention, 4 (14%) decided to leave prematurely. Thus, based on the definition of dropout in this study, an adherence rate of 83% (24/29) was recorded, including 1 withdrawal and 4 dropouts.

Of the 30 caregivers who signed the informed consent form, 1 (3%) did not continue the baseline assessment due to difficulties in arranging an electronic device, such as a laptop or tablet, to continue the study. In addition, of the remaining 29 caregivers, 1 (3%) withdrew due to the death of the person with dementia, and 4 (14%) dropped out citing concerns that the study was too time-consuming or emotionally challenging or added an extra burden to their existing caregiving responsibilities. Examples of reasons for dropout included the following:

I wasn’t that fond of it. It makes you depressed, all that thinking about the past and stuff. It’s not what I expected.

ACT-IC015; aged 63 years; male

I don’t want to dwell on it, don’t want to learn to think differently either, I find that scary.

ACT-IC018; aged 51 years; female

No differences were identified between caregivers who dropped out and those who completed the study. Of the 24 participants who completed the study, most were female (18/24, 75%), and the hours of caregiving were often reported to be >15 per week, ranging from 3 to 20. The duration of dementia (ie, time since diagnosis) was, on average, 4.1 (SD 2.7) years, with a higher prevalence of Alzheimer (12/24, 50%) followed by vascular dementia (6/24, 25%). Further sociodemographic characteristics of the caregivers are shown in Table 2.

**Table 2:** Overview of the sample characteristics (N=24).

Characteristic			Values
Age (y), mean (SD)			62.5 (13.1)
**Sex, n (%)**			
	Female	18 (75)	
	Male	6 (25)	
**Kin relationship, n (%)**			
	Spouse	14 (58)	
	Child	9 (38)	
**Ethnicity, n (%)**			
	Non-Hispanic White	24 (100)	
**Education, n (%)**			
	Completed college	15 (62)	
**Employment status, n (%)**			
	Currently employed	10 (42)	
**Living arrangement, n (%)**			
	Living with care recipient	17 (71)	
**Hours of caregiving per week, n (%)**			
	≥15	15 (62)	
**Dementia diagnosis, n (%)**			
	Alzheimer disease	12 (50)	
	Vascular	6 (25)	
	Young onset dementia	1 (4)	
	Frontotemporal	1 (4)	
	Parkinson disease	2 (8)	
	General (diagnosed, not specified)	2 (8)	
Duration of dementia (y), mean (SD)			4.1 (2.7)

### Results of PPQ

#### Overview

**Figure 3:**
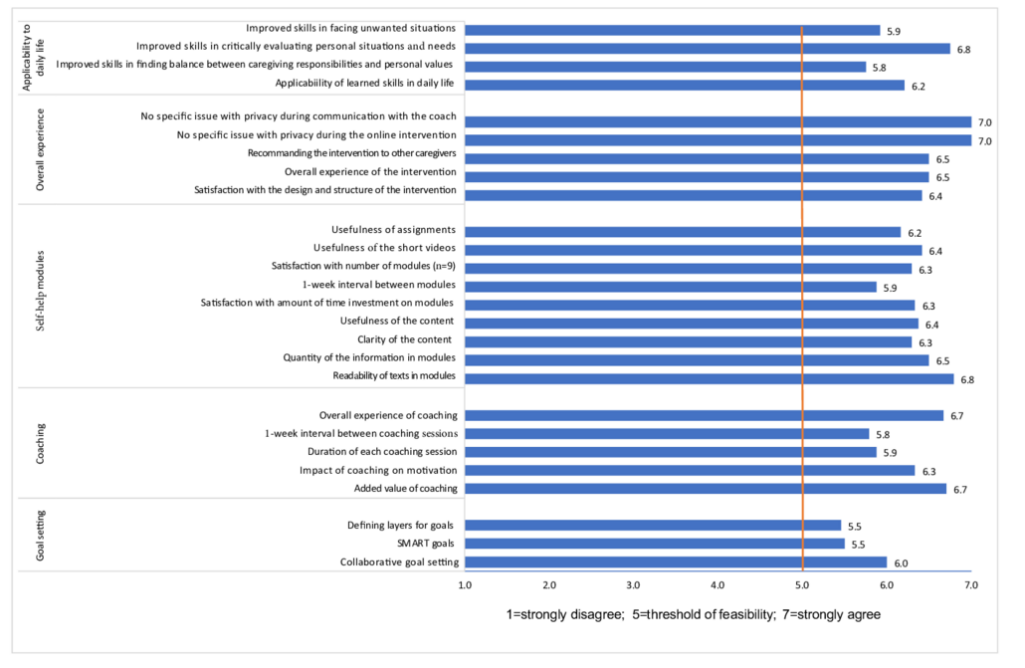
Average scores on each item of the Program Participation Questionnaire. SMART: specific, measurable, attainable, realistic, and time-orientated.

The total score on the PPQ ranged from 90 to 182. The average total score on the PPQ of the 24 caregivers who completed the intervention was 163.4 (SD 22.3). On average, family caregivers gave all items a score of at least 5 (mean 6.29, SD 0.46), with a total median score of 172, indicating high perceived feasibility and acceptability (Figure 3).

The quantitative results of the PPQ on intervention feasibility and acceptability were consistent with postmodule self-report acceptability as well as with deductive qualitative content analysis of the field notes.

Qualitative feedback informed the feasibility and acceptability of the intervention components, specifically (1) overall experience, (2) goal setting and value identification, (3) web-based self-help ACT modules, and (4) coaching. In addition, a semistructured interview was conducted with the motivational coach to gain more insights into study feasibility for the interventionist.

#### Overall Experience

Overall, participants found the intervention’s design comprehensive, logically structured, and strongly interconnected, “like a string of chain,” emphasizing that removing a single component would disrupt the functionality of the intervention. Enhanced adaptability to the caregiving role and improved ability to focus on meaningful life values while providing care for people with dementia were noted as valuable components in addressing the balance between caregiving and self-care. Caregivers reported that their enhanced psychological flexibility, achieved through ACT principles, supported their resilience and also positively influenced people with dementia, describing it as a “win-win” situation:

It was aligned, enriching, confronting, and educational. Without a coach, it would have been easier to let things slip. Providing care becomes much more sincere and high quality by being mindful. You can only do that if you have space for it, so you have to let go of your need for control. It starts with yourself, and you can apply it everywhere, that is a relaxing feeling. I learned to deal with myself in a different way. I could respond more calmly. I wasn’t overwhelmed and overstimulated; it had such a positive effect on the person with dementia.

ACT-IC 002; aged 47 years; male

Caregivers suggested the inclusion of a psychoeducational module specifically addressing the application of ACT to the unique challenges caregivers face in dementia care. Such a module may support contextualizing ACT and enhance the understanding of the metaphors used within the intervention, making it more relevant and applicable to the unpredictable and progressive nature of the condition. In addition, some caregivers expressed the desire to have ongoing access to the modules or receive a printed booklet containing all the modules, allowing them to reference the material even after the study. While acknowledging that the intervention was confronting and required more time than initially anticipated, participants still expressed their recommendation of the intervention to other caregivers.

#### Goal Setting and Value Identification

Family caregivers expressed that setting goals before participating in the intervention was essential for their engagement with the program and establishing a road map toward desired behavior change. Formulating SMART goals made the process more tangible for participants and stimulated a sense of purpose during the intervention, which led individuals to plan and adapt self-management and personal values to their caregiving responsibilities. Most caregivers often emphasized the importance of receiving guidance in value identification, prioritizing goals, breaking value-based actions down into manageable steps, and adjusting goals when necessary:

Providing guidance is important because then I receive a kind of confirmation that I should do it as well. Otherwise, it remains so open-ended. The coach allowed me the freedom to set my own goals and provided guidance when needed. Setting goals and timeframes helped me avoid feeling guilty about taking time for myself.

ACT-IC012; aged 65 years; female

#### Goal Attainment

Participants had mixed experiences when it came to engaging with the action list, which included layers of goal attainment, as well as when they were asked to self-report their progress on their goals during meetings with the coach. Some caregivers found the action list to be beneficial for adapting their goals to better align with the changing demands of caregiving, enhancing their ability to navigate through personal values and caregiving responsibilities effectively. Nonetheless, some caregivers encountered challenges in both accomplishing their goals and accurately assessing their level of achievement. Furthermore, a desire to modify their goals was also noted among some participants. Factors that influenced the attainment of goals primarily included caregiving demands such as the inability to leave the care recipient alone, their own health conditions (eg, physical pain), and time limitations. These factors often resulted in adjustments to the goals throughout the intervention. In addition, a shift in focus to a different set of values was another factor that influenced goal adjustment.

Reduced engagement with the action list or failure to attain goals at a desirable level for some caregivers caused feelings of anxiety or increased burden when reporting to the coach:

I had the tendency to give socially desirable answers when the coach asked if I had worked on my goals. I would be asked if I had gone cycling, but I can’t leave my partner alone. So, I do want to take more time for myself, but the situation doesn’t always allow me to engage in outdoor activities.

ACT-IC 009; aged 74 years; female

According to the coach, using the SMART framework for goal setting was valuable for structuring collaborative goal-setting sessions. However, adjusting goals to account for comorbidities and caregiving demands presented challenges at times in providing guidance, particularly within limited time frames.

#### Web-Based Self-Help ACT Modules

##### Content

The content of the web-based self-help ACT modules was reported mainly as “easy to follow” and helped caregivers in adapting their coping strategies, acquiring new skills to navigate challenges, cultivating mindfulness of the present moment, and reframing their perspective to align with their life values. According to caregivers, following ACT principles in parallel with goals empowered them to gain more insights into psychological flexibility, enabling them to take practical steps toward living in accordance with their values:

I went out of my comfort zone...I usually think that if you want something, you’ll just do it, but it (i.e., ACT) was truly an eye-opener. I was constantly realising I need to do this, oh yes, I need to pay attention to that as well, of course. Those moments of realisation, I found them very valuable.

ACT-IC 004; aged 52 years; female

After completing each module, participants provided self-report feedback and rated the usefulness, stressfulness, and applicability of the content on a Likert scale ranging from 1 (*strongly disagree*) to 7 (*strongly agree*). All participants (24/24, 100%) completed all modules along with the self-report questionnaires. According to their feedback, the web-based self-help modules were generally perceived as useful, with a mean score of 5.6 (SD 0.2; range 5.4-6.1), and applicable to daily life, with a mean score of 5.2 (SD 0.5; range 4.4-5.9). The stressfulness of the content was rated with a mean score of 3.5 (SD 0.8; range 2.4-4.4). Among the various components, the “self as a context” component was identified as the most useful yet one of the most stressful modules after “Acceptance” (Multimedia Appendix 8).

Overall, participants expressed that the ACT modules enabled them to “change attitude,” which was necessary for openness and developing skills that are “potentially applicable” in everyday life. However, taking perspective did not always result in actual changes in behavior in everyday life for some (older) caregivers. The most frequently reported barrier to applying new skills “in the situation” was the role of habits. Caregivers often highlighted that implementing new ACT skills took time to “sink in,” particularly due to habitized responses to specific cues that developed through years of repetition:

You’ve been doing things your whole life, often unconsciously, and now you’re becoming aware of things. Becoming aware and then changing your behavior takes time.

ACT-IC001; aged 65 years; female

In difficult situations, it’s not so easy to implement everything you know. You know it now, but it can still be challenging to apply in the situations.

ACT-IC006; aged 71 years; female

With a certain age and lifestyle, it’s a significant adjustment, not easy, but the adaptations are necessary.

ACT-IC011; aged 79 years; male

According to the coach, extending the completion time frame to 12 weeks allowed caregivers more time and space to learn the materials. However, given the time required to acquire new skills, extending the time frame to >12 weeks could also be beneficial.

##### Format

Most participants found 1 module per week to be suitable, with some suggesting that 2-week intervals could also work. In total, 29% (7/24) of the caregivers required >9 weeks to complete all 9 modules. Caregivers found the web-based format beneficial as it eliminated the need for additional travel time, making it convenient to integrate the intervention into their daily routines. In addition, the convenience of the self-help modules was appreciated, which allowed caregivers to review the material and take notes at their own pace, offering an opportunity to consider personal preferences:

I have difficulty remembering certain things (concerning the intervention material). I have to reread things to apply them properly.

ACT-IC001; aged 65 years; female

All participants (24/24, 100%) completed the intervention using computers or laptops with internet access. Regarding the experience of the web-based format, caregivers expressed that the presence of a coach supported them with navigating through the web setup. The combination of video, text, and assignments was expressed as useful to comprehend ACT exercises and metaphors. However, some caregivers chose to review the material either by accessing the web application on their mobile phones or offline by printing the material rather than logging into their accounts via their computers:

I printed it out so I could read it and write down my thoughts and notes. I prefer paper over online, which might have to do with my age.

ACT-IC006; aged 71 years; female

According to the coach, addressing technical difficulties provided the necessary support for participants with lower digital literacy and prevented dropout due to technical issues.

#### Coaching

Overall, participants found the coaching sessions aligned, enriching, and constructive. The supportive listening, availability of the coach through multiple channels (video call, phone call, and email) during coaching, technical support for the web-based module, feeling safe with privacy on the web, and flexible rather than fixed coaching appointments were particularly appreciated. Caregivers reported that receiving personal feedback enhanced their active engagement with the entire intervention. This engagement, in turn, increased their motivation to learn new perspectives presented by the ACT modules and strengthened their commitment to achieving their goals. The contact with the coach through digital means was mentioned as added “accountability” and a suitable modality, particularly for those who preferred direct contact with health care professionals.

### From the Coach’s Perspective

The weekly coaching sessions revealed several prominent barriers faced by caregivers, including technological, intrinsic, and extrinsic challenges. Technological barriers centered on issues related to digital literacy, limited access to digital devices, and a lack of self-efficacy in technology use. The coach addressed these concerns by offering supplementary guidance, providing the option to print materials for offline use, and enabling access to the intervention through a mobile-friendly web application. Intrinsic obstacles included motivational struggles, physical discomfort, and reduced sensory abilities, which influenced the caregivers’ willingness to implement new ACT strategies or attain their goals. The coach navigated these barriers through motivational conversations, supportive listening, and adaptable goal setting to accommodate individual preferences and limitations. Extrinsic barriers included time constraints, demanding caregiving responsibilities, and the evolving condition of the care recipient, leading to fluctuations in the caregivers’ schedules and diaries.

Regarding the nontherapeutic nature of the coaching, the coach expressed that “caregivers just needed to be heard.” However, providing motivation without knowing the context was often not possible, resulting in the duration of sessions exceeding the planned 20 minutes. Although the longer calls were still feasible for the coach, providing flexible availability was not always easy to adjust to daily work responsibilities. Furthermore, the coach noted that conducting data collection fully over the web and providing technical support posed occasional challenges that sometimes required contacting third parties and could result in delays.

## Discussion

### Principal Findings

This feasibility study was conducted in response to the demand for interventions that are both more scalable and personalized for family caregivers of people with dementia. The findings of this study revealed that caregivers acknowledged the beneficial impact of psychological flexibility. This recognition resulted in perceived positive effects not only in self-management and caregiving quality but also in the applicability of ACT in noncaregiving situations in daily life. This outcome is in line with that of previous research that ACT can be transdiagnostic in addressing psychological flexibility in a heterogeneous sample of caregivers who provided care for individuals at various stages and with various types of conditions, including dementia [46,47]. The findings of this study revealed a high adherence rate of 83% (24/29) at the postintervention measurements, which exceeded the average adherence rate of 57% found in self-guided ACT interventions [48], 73% found in self-help interventions [24], and 69% found in internet-based treatments for the general population [49]. High adherence and overall satisfaction are particularly important findings as family caregivers of people with dementia have the highest dropout rates and reported problems with access and usability of eHealth interventions [4].

The aim of this guided web-based intervention was to broaden the evidence base by exploring practical and conceptual barriers and facilitators influencing feasibility and acceptability. This investigation provides additional insights for refining future interventions and potentially facilitates the implementation of effective controlled trials on a larger scale. This web-based self-help intervention was designed for family caregivers of people with dementia and integrated ACT modules with collaborative goal setting based on personal values and weekly nontherapeutic coaching.

### Personalizing ACT Interventions Through Goal Setting

In line with previous research, our findings showed that collaborative goal setting based on caregivers’ values holds promise as an approach to address the specific needs of caregivers and personalize interventions [36]. In addition, collaborative reflections and guidance played a crucial role in the process of value, need, and resource identification when setting SMART goals as well as adjusting goals or timings throughout the intervention. This adaptability was perceived as necessary due to the continuously changing care demands and concurrent circumstances for caregivers. Multiple participants who set goals consistent with their originally selected values changed their goals focusing on a different value domain. Consistent with previous research, our findings underscore the significance of maintaining flexibility in future intervention designs, allowing for the selection of personally relevant values and value-focused exercises [50].

From the ACT perspective, values are meaningful qualities that cannot be obtained or finished but rather guide long-term patterns of behavior [11]. Subsequently, acknowledging personal values and nesting specific goals underneath them is more likely to drive effective behavior change in the long term [51]. Thus, the shift in focus to a different value domain may represent a potential intervention effect, enhancing caregivers’ motivation to align their lives with their values. Further investigation is also warranted to closely examine the factors that drive caregivers to modify their value-based goals following their involvement in a value-based intervention. Such an exploration will shed light on whether these changes in goals are motivated by a genuine desire of caregivers to align their lives with their core values.

In the context of personalizing ACT interventions, facilitating skill building and modifying behavioral responses in older adults with regard to potential concurrent challenging circumstances (eg, health conditions) might be especially important [52]. Moreover, technology offers opportunities to personalize future interventions by providing real-time feedback or guidance (eg, using experience sampling methodology and “just-in-time” interventions), promoting engagement with and managing multiple goals over time, simplifying complex goals, and facilitating collaborative or interactive reflections [53]. Personalizing eHealth interventions for future studies is particularly noteworthy as person-centered (family) care emerges as a crucial scope of research in the path of global dementia care [54].

### Utility of ACT for Behavior Change in Caregivers

Our findings suggest that ACT contributes to an increase in perceived psychological flexibility and perspective taking. For some caregivers, increased psychological flexibility might enable them to prioritize focusing on personal growth and self-care as well as embracing challenges over acquiring high goal attainment scores or external validations and motivation (eg, those provided by the coach) [55]. However, perspective taking for some caregivers did not lead to a reported change in behavior.

For some caregivers, defining an appropriate goal or putting goals into action was challenging due to concurrent circumstances (eg, comorbidities). Family caregivers noted that, despite their change in attitude through practicing ACT, reconfiguring their habitual responses based on the new insights from ACT remained challenging in certain situations. It was difficult to adopt a new mindset and avoid reacting impulsively, especially in response to the unpredictable symptoms of the person with dementia. This suggests that behaviors may be triggered by contextual cues rather than being solely the result of mindful attention or personal willpower [56].

This finding is in line with that of previous research highlighting that habits might impact the relationship between attitudes and behavior, resulting in the regulation of desirable behavior change in the long term. Therefore, a change in attitude in caregivers, although crucial for behavior change, might be influenced by situational factors (eg, behavioral symptoms of the person with dementia), potentially making them less reliable predictors of behavior [56]. Addressing habit formation in the context of dementia caregiving is particularly noteworthy as behavioral symptoms and the deteriorative nature of dementia might lead to an unstable, impulsive, unpredictable, and stressful context for caregivers.

Future design of behavior change interventions can target habit formation and the interplay between attitudes and habits for the consolidation of effective upskilling and long-term behavior change in caregivers. This effort may involve developing ACT interventions with additional modules, such as behavior modification strategies for managing disruptive behaviors [13], tailored mindfulness, or habit reversal training [57]. In this endeavor, specific attention to discovering intervention mechanisms may benefit from the use of experience sampling methodology as a quantitative approach [58].

In addition, several participants in this study suggested the inclusion of an explicit module addressing the specific context of dementia and dementia caregiving. Accordingly, a module designed to incorporate ACT principles and provide guidance on managing dementia-specific challenges, such as effectively managing repetitive questions from people with dementia, could significantly enhance the applicability of ACT to their unique situation. This result is in line with those of previous research highlighting that caregiving itself is a natural value for caregivers [59]. The future development of ACT interventions for caregivers can be centered on values associated with caregiving (ie, maintaining care and improving the relationship with the person with dementia) while allowing for variations among different caregiver profiles based on their individual value preferences [28]. Further empirical research is now needed for further evidence-based understanding of the impact of value commitment on caregiver and care recipient outcomes.

### Guided Self-Help Interventions for Caregivers

In the context of caregiving, adhering to web-based self-help interventions and incorporating them into everyday life commitments is thought to be improved by embedding more personal retention approaches (eg, telephone calls), provision of flexibility, personalization, scalability, and guidance [4,14,23]. Our study showed that a web-based self-help ACT intervention with synchronous motivational coaching was highly feasible and acceptable for family caregivers, suggesting that ACT and skill building can be learned through self-help [24].

It is important to recognize the coach’s significant role in enhancing adherence, motivation, and confidence in technology use as well as fostering a willingness to adopt eHealth among family caregivers of people with dementia. In line with previous research, our findings suggest that providing training on technological features and access to troubleshooting might be beneficial for caregivers with lower digital literacy as, this way, they might be less likely to perceive eHealth as difficult, incompatible, or ineffective. Furthermore, including dedicated coaches for guidance and offering technical support might more likely generate a positive attitude toward eHealth [29].

Although this study provided insights into the role of coaching in maintaining intervention adherence, providing resource-heavy support does not reflect how ACT-based programs are often used at a broader scale and outside the research context [48]. Future designs of personalized and guided eHealth interventions can explore how technology can provide additional support to health care professionals as providers to reduce personal resources and also facilitate the design of effective and implementable interventions on a large scale. Subsequently, guided self-help interventions for family caregivers can evaluate the effects of varying degrees of contact, allowing for the realization of what is the minimum amount of contact that should accompany self-help to obtain the maximum benefit [60]. To support effective, acceptable, and sustainable interventions, future approaches could involve caregivers in the process of designing interventions to capture both the context and dementia-specific needs of caregivers to be tested in larger samples [9].

### Adapting ACT to a Web-Based Format for Older Adults

Although websites are the most common way of delivering web-based ACT interventions and are typically more feasible for researchers to build [61], our results showed the preference of some participants to use the intervention via their mobile phones (web application). Considering that mobile apps are more accessible and are the most commonly accessed method of self-help, future research can consider ACT-based apps for family caregivers to address the discrepancy between the ACT literature and real-world practice [48]. This technological development is noteworthy for bridging the gap to make therapy available, accessible, and affordable for larger populations of family caregivers who do not need heavy support [62]. Implementing interventions in the real world is essential for caregivers as, despite the need for behavior change interventions, most feasible and effective eHealth interventions for family caregivers of people with dementia are not yet ready for implementation and, thus, not implemented in the real world [63].

### Strengths and Limitations

This study introduced a blended intervention to address the crucial need for effective interventions in dementia caregiving by exploring the feasibility of a web-based ACT intervention tailored for this population. The strengths of this study lie in its contribution to the need for further evidence-based interventions in the area of ACT and family care, paving the way for future controlled trials and intervention refinement. Additional strengths include high adherence rates, flexibility in delivering the intervention to a diverse population of caregivers of people with dementia, and shedding light on barriers and facilitators that family caregivers experienced over the course of the guided web-based self-help intervention. The mixed methods approach combined quantitative adherence rates and qualitative caregiver feedback for a comprehensive understanding of the intervention.

Nevertheless, this study might be influenced by potential biases, and when interpreting the findings, it is crucial to acknowledge its limitations. Although conducting feasibility studies before an RCT can ensure the design of studies with a higher likelihood of success, the small sample size in this study necessitates caution when generalizing the results to a larger population of caregivers of people with dementia. The study sample size was relatively small (N=30), which may limit the generalizability of the findings. Larger samples are required to draw more robust conclusions and account for potential individual differences among caregivers. In addition, the study duration was 9 weeks, and it lacked a control group, which made it challenging to determine whether the observed positive outcomes were solely due to the intervention or whether other factors may have contributed to the results. Conducting larger studies with long follow-up assessments and including a control group would provide a better basis for assessing this guided web-based intervention.

Participation in the study was voluntary and occurred over the web. Thus, individuals who chose to participate may not constitute a fully representative sample, potentially skewing toward those who are more technologically savvy with higher levels of education and greater familiarity with technology. It is crucial to acknowledge this selection bias and consider generalizability concerns when interpreting the findings. ACT principles underscore that individuals do not always have the autonomy to select the content of any given situation. To convey this concept, ACT uses metaphors that may necessitate intellectual engagement and abstract reasoning [10]. Considering the above-average level of education among the study participants and the availability of a trained coach for questions, our findings may not be broadly generalizable to caregivers with lower digital literacy or a lower educational background.

We used a guided ACT-based intervention blended with other non-ACT complementary techniques (eg, goal setting) and components (eg, weekly coaching), which might have had therapeutic effects. Specifically, some caregivers received more than the anticipated 20 minutes of coaching, leading to an unequal distribution of coaching among participants. This variability, as well as the use of self-report questionnaires, could potentially influence the generalizability of the study findings. In addition, most caregivers were at an early stage of caregiving and cared mainly for people with Alzheimer disease, limiting the understanding of how the intervention works across different contexts and stages of dementia caregiving.

### Conclusions

This study was conducted to evaluate the feasibility and acceptability of a guided web-based self-help ACT intervention for family caregivers of people with dementia. The high adherence rate and positive feedback from caregivers indicate the intervention’s feasibility and acceptability. The findings suggest that family caregivers can learn ACT principles and use them to enhance their psychological flexibility through self-help. Moreover, personalizing the intervention through collaborative goal setting based on individuals’ values was found to be promising for addressing the specific needs of caregivers. The findings also suggest that ACT can be adapted to a web-based format, increasing accessibility and scalability for a diverse and large sample of caregivers. However, the absence of a control group and small sample size limit the drawing of definitive conclusions. Some caregivers faced challenges in translating new skills into behavior change due to habitual responses. Larger controlled trials are needed to validate the feasibility in a more diverse caregiver sample and determine the effectiveness of ACT-based interventions in this population. This study highlights the need for future interventions to address habit formation and the interplay between attitudes and habits in unpredictable and continuously changing caregiving contexts. In addition, exploration of the impact of value-based behavior on caregiver and care recipient outcomes should be considered in further research. It is hoped that the results of this feasibility study will pave the way for future effective controlled trials and the implementation of evidence-based research in real-world settings.

## Appendix

Multimedia Appendix 1 CONSORT-EHEALTH (Consolidated Standards of Reporting Trials of Electronic and Mobile Health Applications and Online Telehealth) checklist V 1.6.1.

Multimedia Appendix 2 An overview of family caregivers’ potential personal values.

Multimedia Appendix 3 Steps of collaborative goal setting.

Multimedia Appendix 4 The interface of the website used to deliver the intervention.

Multimedia Appendix 5 An overview of the weekly coaching questions.

Multimedia Appendix 6 An overview of the Program Participation Questionnaire.

Multimedia Appendix 7 Semistructured interview with the coach.

Multimedia Appendix 8 An overview of the acceptability of the self-help modules.

## Abbreviations

ACT: acceptance and commitment therapy
CONSORT: Consolidated Standards of Reporting Trials
PPQ: Program Participation Questionnaire
RCT: randomized controlled trial
SMART: specific, measurable, attainable, realistic, and time-orientated
