# Supplementary material for: A Web-Based Intervention Based on Acceptance and Commitment Therapy for Family Caregivers of People With Dementia: Mixed Methods Feasibility Study
Source: JMIR Aging. 2024 Apr 4;7:e53489. doi: 10.2196/53489 (PMC11027053; doi:10.2196/53489)
Supplement: Multimedia Appendix 2 [file aging_v7i1e53489_app2.docx]

**Appendix 1**

*An overview of family caregivers’ potential personal values*

| Self-development | Learning, training or improving skills, finally start a long-awaited activity, raising knowledge about a particular concept, explore something that has been a long-time interest, educating yourself, art, creative expression, and aesthetics |
| --- | --- |
| Physical self-care | Exercise, physical activity, increasing inside or outside mobility individually or in a group, body movement or any type of sports such as yoga or walking |
| Social life | Spending time with friends, communities, neighbours, social activities, talking to people with shared interests, making friends or meeting new people, group actions |
| Recreation | Leisure activity, fun, any kind of hobby, short trips in nature, relaxation, movies, music, photographing, reading novels and stories, cooking, or any other activity that brings joy and emotional satisfaction |
| Caregiving | Improving balance of caregiving responsibilities, care-related time- and self-management, spending quality time with the care recipient |
| Health | Self-care, diet, skin care, sleep, and/or any kind of medical support to achieve a greater sense of health and well-being or reduce pain, starting a new healthy habit/routine and behaviour, increasing mental health, follow up or check up on previous decease, visiting a chiropractor, optometrist, etc |
| Work | Starting or improving skills that help with employment, retirement or any type of job or profession-related responsibilities |
| Spirituality | Pray, worship, religious studies or spiritual activity that may be associated with peace of mind |
| Family relation | Spending quality time with other family members, children, siblings, cousins, spouse, partner, couples or any family-related activity |
| Citizenship | Moving forward with legal processes of selling/buying properties, registering/cancelling selective services |
