# Supplementary figures and images for: A Web-Based Intervention Based on Acceptance and Commitment Therapy for Family Caregivers of People With Dementia: Mixed Methods Feasibility Study
Source: JMIR Aging. 2024 Apr 4;7:e53489. doi: 10.2196/53489 (PMC11027053; doi:10.2196/53489)

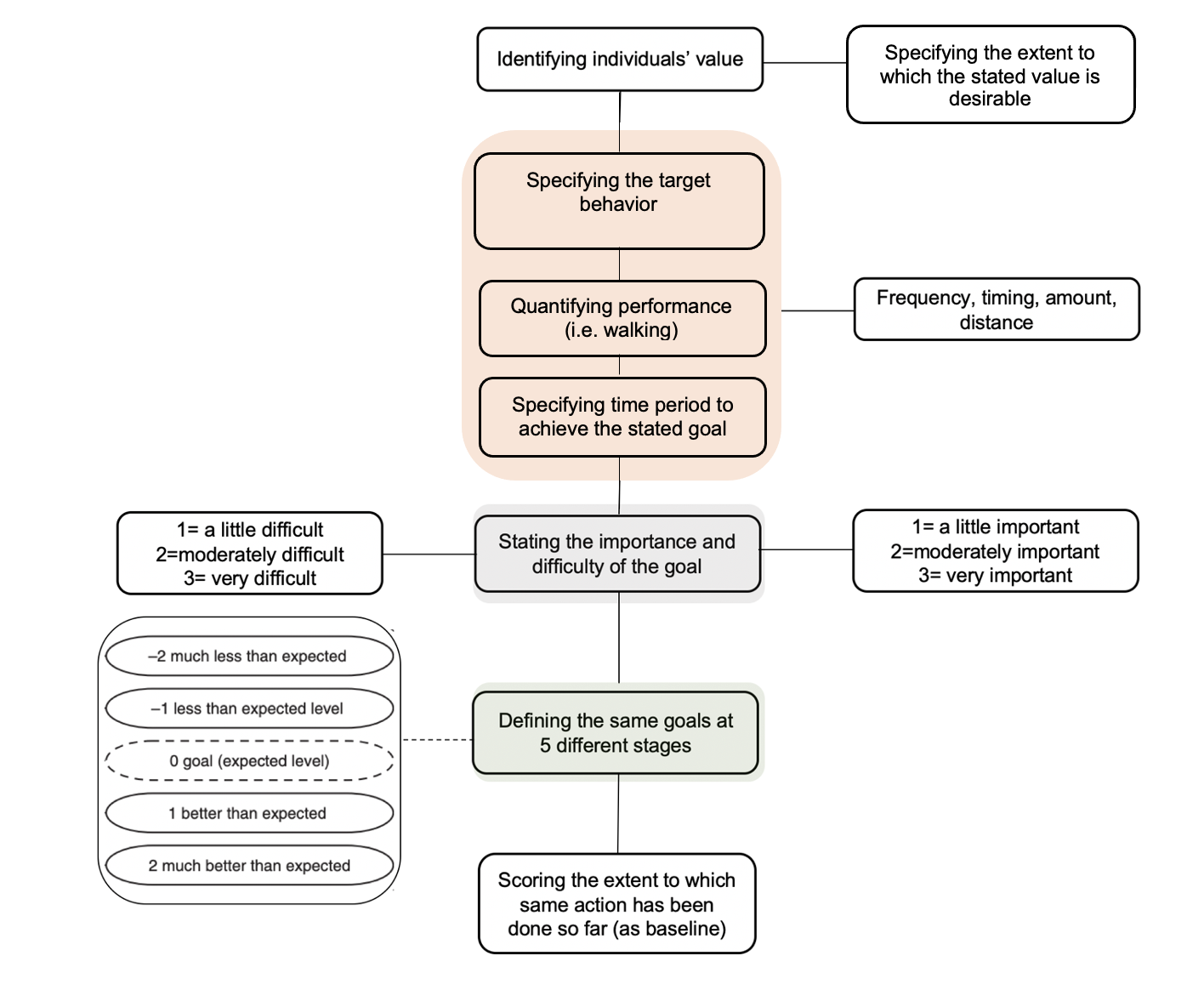

Supplement: Multimedia Appendix 3 [file aging_v7i1e53489_app3.docx]

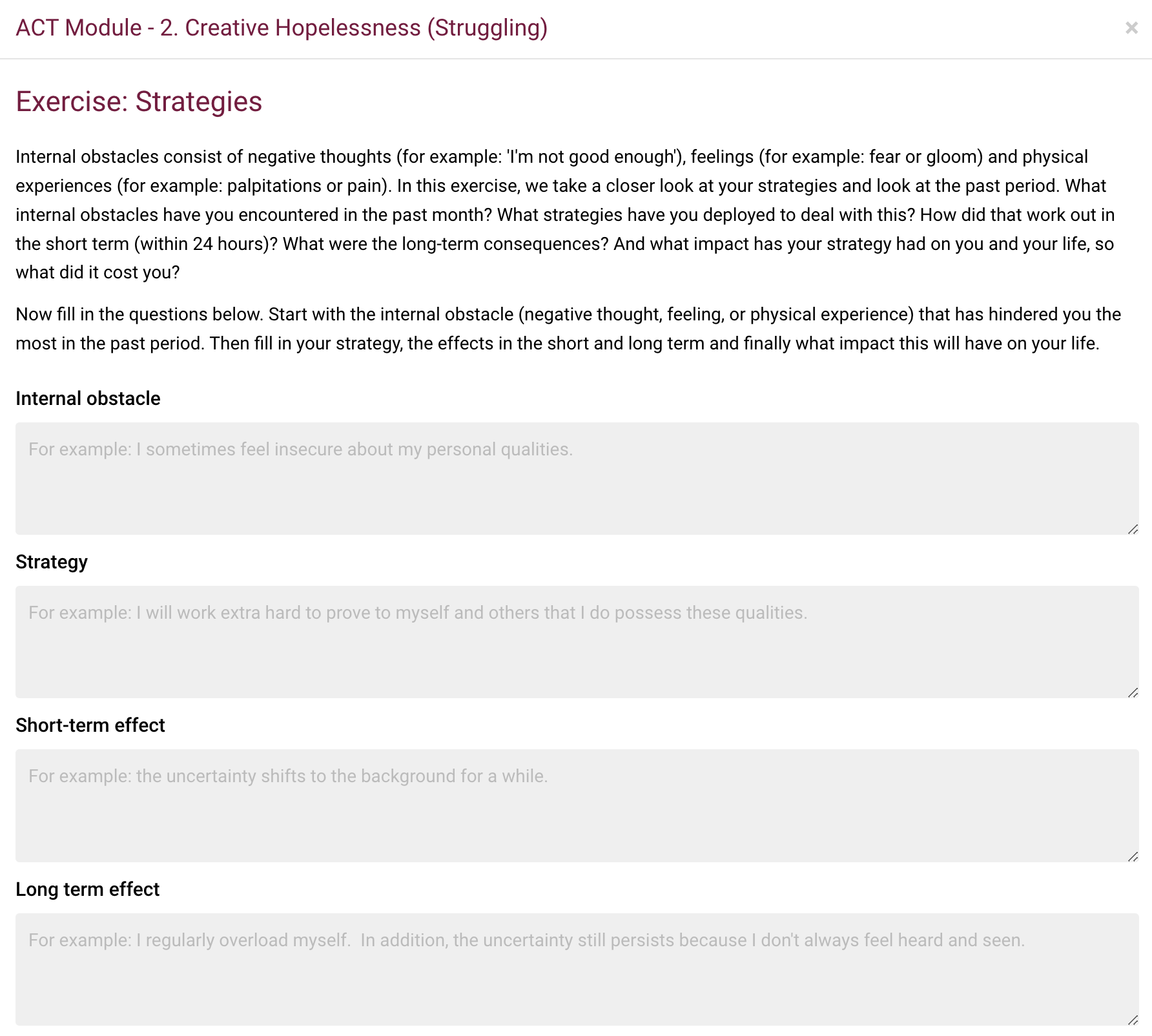


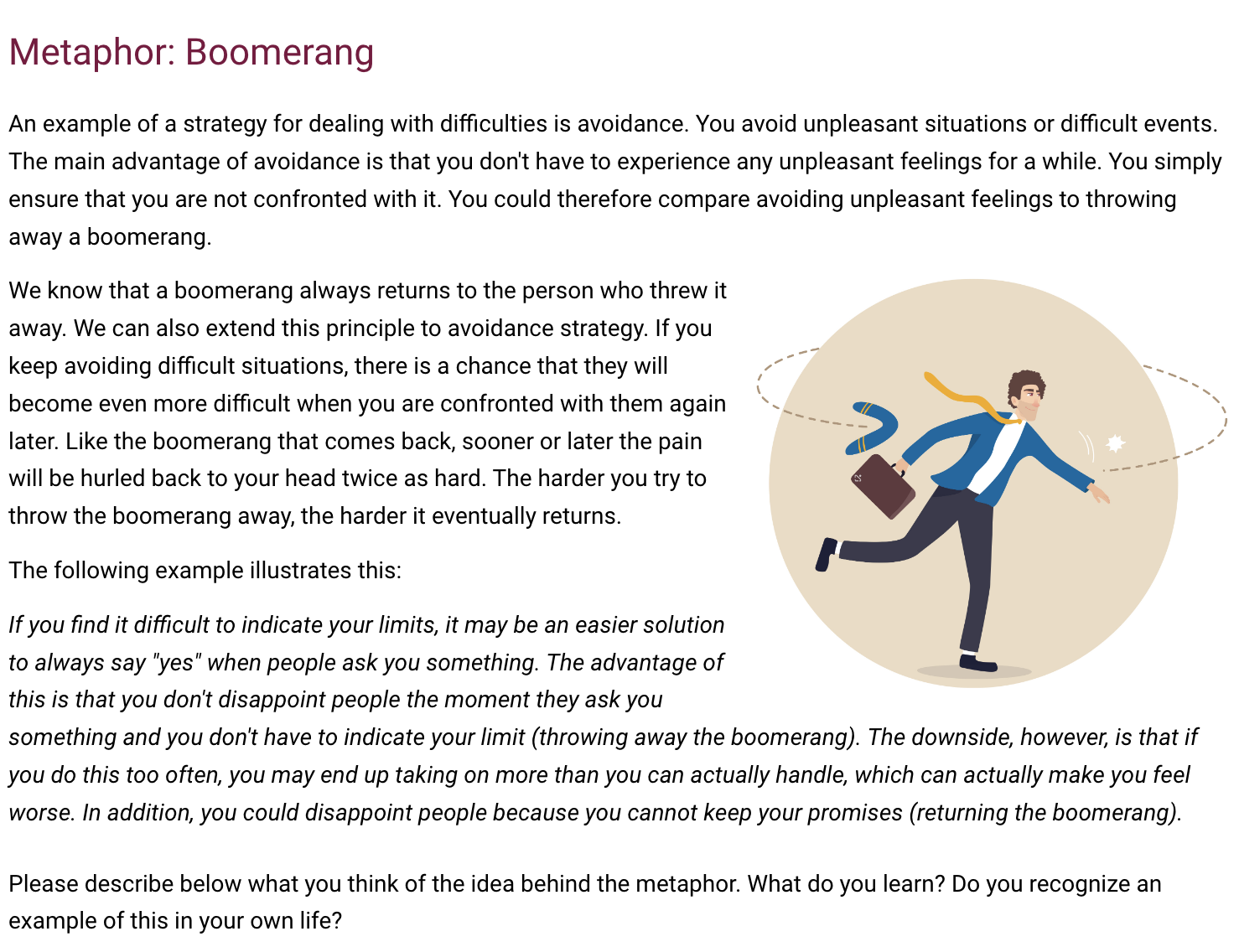


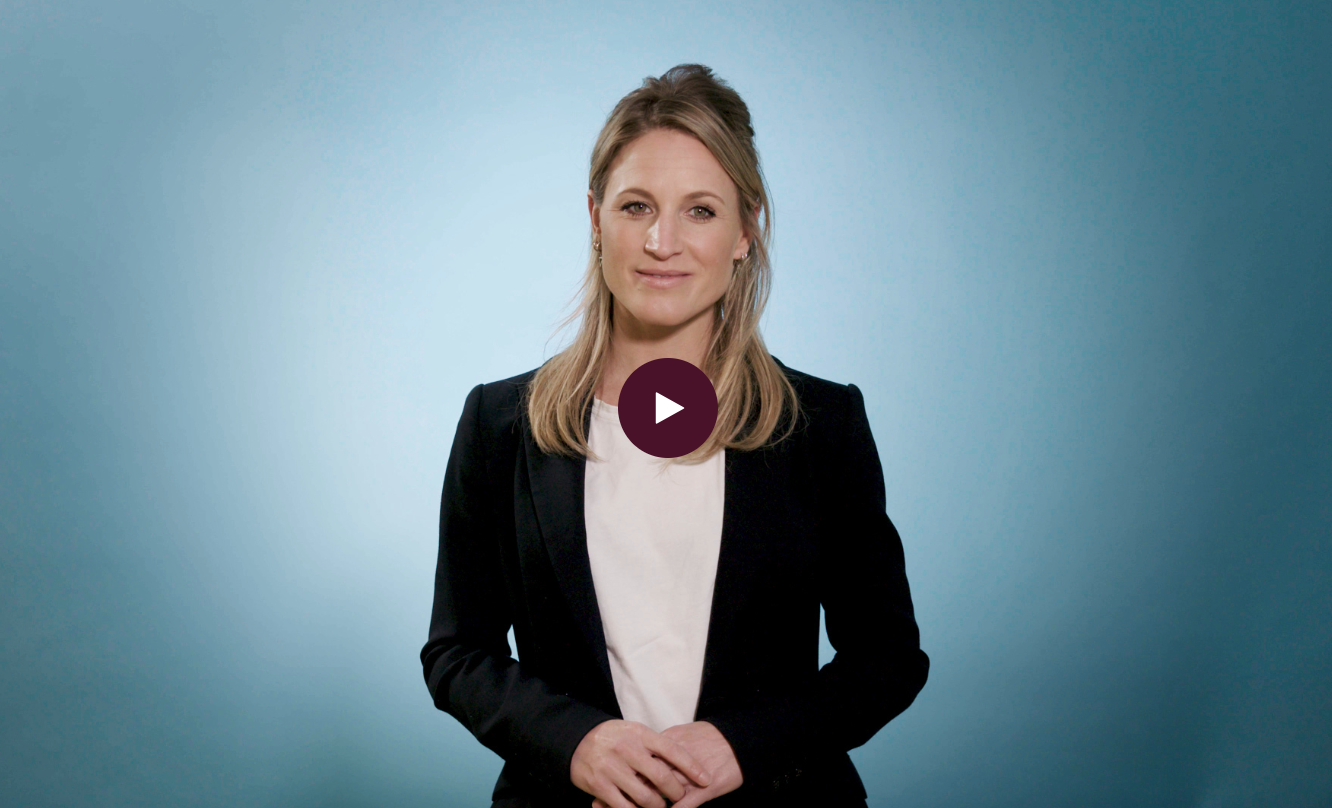

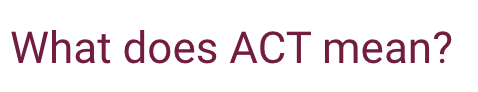


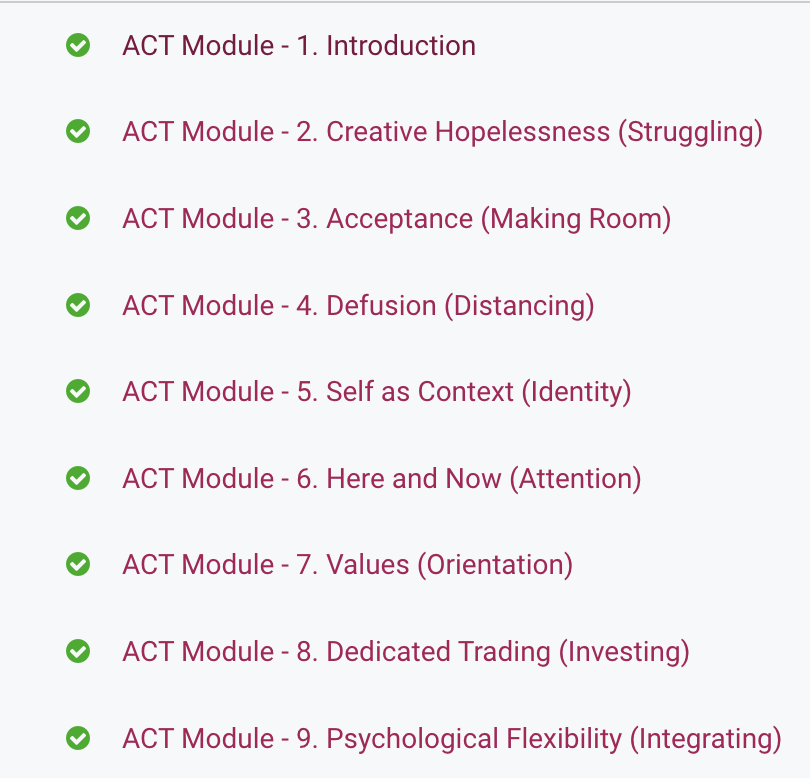

Supplement: Multimedia Appendix 4 [file aging_v7i1e53489_app4.docx]
