# Supplementary material for: A Web-Based Intervention Based on Acceptance and Commitment Therapy for Family Caregivers of People With Dementia: Mixed Methods Feasibility Study
Source: JMIR Aging. 2024 Apr 4;7:e53489. doi: 10.2196/53489 (PMC11027053; doi:10.2196/53489)
Supplement: Multimedia Appendix 5 [file aging_v7i1e53489_app5.docx]

| *Motivational coaching during the “ACT-IC” intervention* | Reflection |
| --- | --- |
| 1. General experience of caregivers with the intervention/modules. |  |
| 2. Was the caregiver sufficiently able to complete the module during the previous week? Please explain. |  |
| 3. To what extent was the caregiver motivated last week? If there were changes in motivations, please elaborate on the factors that played a role. |  |
| 4. Please describe what (technical) problems arose during using the website and going through the module? |  |
| 5. To what extend the caregiver was able to work on his/her own goal? |  |
|  |  |
