# Supplementary material for: A Web-Based Intervention Based on Acceptance and Commitment Therapy for Family Caregivers of People With Dementia: Mixed Methods Feasibility Study
Source: JMIR Aging. 2024 Apr 4;7:e53489. doi: 10.2196/53489 (PMC11027053; doi:10.2196/53489)
Supplement: Multimedia Appendix 6 [file aging_v7i1e53489_app6.docx]

1= Strongly disagree

7= Strongly agree

| *Participation in the “ACT-IC” intervention* |  | | | | | | | Reflection |
| --- | --- | --- | --- | --- | --- | --- | --- | --- |
| 1. I enjoyed the collaborative goal-setting with the motivational coach | 1 | 2 | 3 | 4 | 5 | 6 | 7 | *Why/why not?*  *- Possible points for improvement?* |
| 2. The action list for my goals helped me to structure a way to change my undesired situations | 1 | 2 | 3 | 4 | 5 | 6 | 7 | *Why/why not?*  *- Possible points for improvement?* |
| 3. I think the weekly coaching was a good addition to the program | 1 | 2 | 3 | 4 | 5 | 6 | 7 | *Why/why not?*  *- Possible points for improvement?* |
| 4. Weekly talk to the coach motivated me to follow my goals | 1 | 2 | 3 | 4 | 5 | 6 | 7 | *- Possible points for improvement?* |
| 5. I found the duration of the coaching sessions (~20 minutes) sufficient | 1 | 2 | 3 | 4 | 5 | 6 | 7 | *- - Shorter or longer?* |
| 6. I think one week interval between coaching sessions was enough | 1 | 2 | 3 | 4 | 5 | 6 | 7 | *- Shorter or longer?* |
| 7. I made use of: |  |  |  |  |  |  |  |  |
| 7a. Conversations with the coach | 1 | 2 | 3 | 4 | 5 | 6 | 7 | *- Why/why not?*  *- What did/didn't appeal to you in this?* |
| 7b. The step-by-step plan toward my goals | 1 | 2 | 3 | 4 | 5 | 6 | 7 | *- Why/why not?*  *- What did/didn't appeal to you in this?* |
| Open reflection |  |  |  |  |  |  |  | *- How did you find the intervention?*  *- Did you struggle with or miss any web features?*  *- Would you delete any certain features? Which/why?* |
| 8. I could read the text on the website well | 1 | 2 | 3 | 4 | 5 | 6 | 7 | *- points regarding text size, contrast, amount?* |
| 9. I liked the amount of information offered per module | 1 | 2 | 3 | 4 | 5 | 6 | 7 | *- Points regarding length of the video, amount of text in the introduction?* |
| 10. I found the content of the modules clear and easy to understand | 1 | 2 | 3 | 4 | 5 | 6 | 7 | *- Easy to understand/follow?*  *- Why/why not?* |
| 11. I found the modules useful | 1 | 2 | 3 | 4 | 5 | 6 | 7 | *- Why/why not?*  *- Possible points for improvement?* |
| 12. I liked the amount of time I spent on each module | 1 | 2 | 3 | 4 | 5 | 6 | 7 | *- How much time did you spend per module?*  *- What did you spend the most time with?*  *- Would you have preferred to spend more/less time on it?* |
| 13. I found one week interval between modules enough | 1 | 2 | 3 | 4 | 5 | 6 | 7 | *- Possible points for improvement?* |
| 14. I liked the number of modules (9) | 1 | 2 | 3 | 4 | 5 | 6 | 7 | *- More or less?* |
| 15. I made use of: |  |  |  |  |  |  |  |  |
| 15a. The introductory videos | 1 | 2 | 3 | 4 | 5 | 6 | 7 | *- Why/why not?*  *- What did/didn't appeal to you in this regard?* |
| 15b. The assignments | 1 | 2 | 3 | 4 | 5 | 6 | 7 | *- Why/why not?*  *- What did/didn't appeal to you in this regard?* |
| Open reflection |  |  |  |  |  |  |  | *- Did you find it complete?*  *- Did you miss any features? Suggestions?*  *- Would you delete certain features? Which?* |
| 16. I liked the structure of the modules (action list, step-by-step plan, modules, coaching). | 1 | 2 | 3 | 4 | 5 | 6 | 7 | *Logical structure*  *- Did you follow all the components? Why/why not?*  *- What part(s) of the intervention did you find most helpful or effective and what parts not?*  *- Did you miss any component?* |
| 17. I am generally satisfied with what was offered to me during the intervention | 1 | 2 | 3 | 4 | 5 | 6 | 7 | *- Why/why not?*  *- Possible points for improvement?* |
| 18. I have used the information offered during the intervention in my daily life | 1 | 2 | 3 | 4 | 5 | 6 | 7 | *- Why/why not?*  *- What was that about?* |
| 19. After taking the course, it is easier for me to find the balance between my personal needs and my caregiving responsibilities | 1 | 2 | 3 | 4 | 5 | 6 | 7 | *- Why/why not?*  *- What was that about?* |
| 20. The course helped me to critically evaluate my situation | 1 | 2 | 3 | 4 | 5 | 6 | 7 | *- Why/why not?*  *- Possible points for improvement?* |
| 21. After following this program I know how to deal with unwanted situations more easily in the future | 1 | 2 | 3 | 4 | 5 | 6 | 7 | *- Why/why not?*  *- Possible points for improvement?* |
| 22. I would recommend the program to other carers of people with dementia | 1 | 2 | 3 | 4 | 5 | 6 | 7 | *-Why/why not?*  *- Which aspects in particular?* |
| 23. I experienced privacy issues: |  |  |  |  |  |  |  |  |
| 23a. In general | 1 | 2 | 3 | 4 | 5 | 6 | 7 | *-What did you (dis)like?*  *- What was that about?* |
| 23b. During communication with my coach | 1 | 2 | 3 | 4 | 5 | 6 | 7 | *-What did you (dis)like?*  *- What was that about?* |
| 24. Open reflection  *- Is there anything else you would like to say about using the program or your satisfaction with the program?* |  | | | | | | | |
