# Supplementary material for: A Web-Based Intervention Based on Acceptance and Commitment Therapy for Family Caregivers of People With Dementia: Mixed Methods Feasibility Study
Source: JMIR Aging. 2024 Apr 4;7:e53489. doi: 10.2196/53489 (PMC11027053; doi:10.2196/53489)
Supplement: Multimedia Appendix 7 [file aging_v7i1e53489_app7.docx]

| *Motivational coach of the “ACT-IC” intervention* | 1= Strongly disagree  7= Strongly agree | | | | | | | Reflection |
| --- | --- | --- | --- | --- | --- | --- | --- | --- |
| 1. As a coach, I found the intervention useful | 1 | 2 | 3 | 4 | 5 | 6 | 7 | *- Why/why not?*  *- Possible points for improvement?* |
| 2. I could integrate the course into my work | 1 | 2 | 3 | 4 | 5 | 6 | 7 | *- Why/why not?*  *- What did/didn't appeal to you in this?* |
| 3. I think the course had added value for family caregivers | 1 | 2 | 3 | 4 | 5 | 6 | 7 | *- Possible points for improvement?* |
| 4. I think the course had added value for me as the coach | 1 | 2 | 3 | 4 | 5 | 6 | 7 | *- Why/why not?* |
| 5. I was able to support the participants according to protocol and as planned | 1 | 2 | 3 | 4 | 5 | 6 | 7 | *-* *Points of improvement?* |
| 6. I found the use of coaching beneficial and it was clearly visible within my work with the family caregivers | 1 | 2 | 3 | 4 | 5 | 6 | 7 | *- Points of improvement?* |
|  |  |  |  |  |  |  |  | *Did you find it complete?*  *- Did you struggle or miss any features? Suggestions?*  *- Would you delete any certain features? Which one?* |
| 7. Open reflection  - Is there anything else you would like to say about the use of this intervention or your satisfaction with the program? |  | | | | | | | |
