# Supplementary material for: A Web-Based Intervention Based on Acceptance and Commitment Therapy for Family Caregivers of People With Dementia: Mixed Methods Feasibility Study
Source: JMIR Aging. 2024 Apr 4;7:e53489. doi: 10.2196/53489 (PMC11027053; doi:10.2196/53489)
Supplement: Multimedia Appendix 8 [file aging_v7i1e53489_app8.docx]

| Content of module | Average time spent in each module (ie, log data) | Usefulness, mean (SD) | Stressfulness, mean (SD) | Applicability, mean (SD) | Example of qualitative feedback |
| --- | --- | --- | --- | --- | --- |
| Module 1—an introduction to ACT^b^ | Approximately 20 min, 2 s | 5.5 (1.4) | 2.6 (1.5) | 4.4 (1.4) | - “The anonymous questions in the module removed my boundaries and made me be honest with myself and prompted me to dare to feel what I really feel.” [ACT-IC 026; aged 63 years; female] |
| Module 2—creative hopelessness: reflecting on dysfunctional avoidance strategies for difficult thoughts and feelings | Approximately 32 min, 32 s | 5.7 (0.9) | 4.3 (1.7) | 5.0 (1.1) | - “This Module was eye-opener. It has made me extra aware of my (coping) strategy.” [ACT-IC 005; aged 72 years; male] |
| Module 3—acceptance: making room for accepting unpleasant feelings, acknowledging the potential struggles of caregivers with their negative emotions | Approximately 33 min, 49 s | 5.6 (1.2) | 4.4 (1.5) | 4.8 (1.6) | - “The module was confronting, it holds up a mirror to you.” [ACT-IC 017; aged 69 years; male] - “I knew it, but I had to become aware of it. I shouldn’t invest energy in what I can’t change. On the other hand, I can work on things I can change. That’s where I have influence.” [ACT-IC 027; aged 72 years; female] |
| Module 4—defusion: distancing oneself from difficult thoughts. Individuals might tend to take their thoughts seriously and fuse with them as if their thoughts were truths. | Approximately 32 min, 46 s | 5.4 (1.5) | 3.5 (1.7) | 4.9 (1.5) | - “I found the defusion module very beautiful—thoughts you have been just thoughts. I’m now more aware of ‘oh, that’s just a thought.’ I can detach more from myself and let go.” [ACT-IC 002; aged 47 years; male] |
| Module 5—self as context: creating room for being flexible with self-image. Individuals might have a tendency to define who they are but also who they should or would like to be based on their self-image, and this attitude might be stressful. | Approximately 37 min, 11 s | 6.1 (0.9) | 4.1 (2.0) | 5.3 (1.2) | - “I now see better that behavioural symptoms of my husband play a role in my feeling of failure and how do I deal with that.” [ACT-IC 009; aged 74 years; female] - “I totally understand the content but it will take time to start applying this as I have been doing things in my way for so long.” [ACT-IC 021; aged 73 years; female] |
| Module 6—here and now: paying sufficient attention to the present moment, which is the only moment when we can actually live, act, and experience. Focusing too much on the past (“if only I had...”) or the future (“what if...”) might not always be helpful. | Approximately 37 min, 10 s | 5.7 (1.2) | 2.4 (1.7) | 5.6 (1.2) | - “Living in the here and now is really a challenge for me. I am usually concerned with the future.” [ACT-IC 028; aged 57 years; female] |
| Module 7—values: actively asking whether values are sufficiently present in individuals’ lives and putting them into practice as well as acknowledging things that really matter in one’s own life | Approximately 48 min, 42 s | 5.8 (0.7) | 3.6 (1.7) | 5.5 (1.2) | - “Personally, I was always in the corner of: commitment, quality, discipline, structure. I didn’t know how to handle my own values.” [ACT-IC 001; aged 65 years; female] |
| Module 8—committed action: actively investing in values and translating them into value-based actions. Defining concrete and feasible actions toward values helps individuals live a more meaningful life. | Approximately 45 min, 36 s | 5.9 (0.8) | 3.7 (1.6) | 5.2 (1.3) | - “Enlighted me with awareness that it is about the road and not the end goal. I will put a reminder on my phone.” [ACT-IC 002; aged 47 years; male] |
| Module 9—psychological flexibility: practicing 6 core skills together to gain psychological flexibility and personal resilience | Approximately 32 min, 25 s | 5.9 (0.8) | 2.6 (1.4) | 5.9 (0.6) | - “It makes you more resilient and you can critically assess your own boundaries and how to approach them when dealing with challenging situations.” [ACT-IC 032; aged 61 years; female] - “...it all came together. It is liberating that I can take moments for myself without judgment. It affects my whole life, work, and leisure.” [ACT-IC 028; aged 57 years; female] |

^a^Scores on a Likert scale: 1=*strongly disagree* to 7=*strongly agree*.

^b^ACT: acceptance and commitment therapy.
